# Edwardsiella tarda Bacteremia Arising From Infected Necrotizing Pancreatitis in a Patient With Cirrhosis

**DOI:** 10.7759/cureus.107398

**Published:** 2026-04-20

**Authors:** Reyleen R Loreto, Anjana Reji, Shivani Thoutireddy, Vladimir Valencia, Parul Aneja

**Affiliations:** 1 Internal Medicine, St. Joseph's Hospital - BayCare, Tampa, USA; 2 Internal Medicine, BayCare Medical Group, Tampa, USA; 3 Infectious Disease, St. Joseph's Hospital - BayCare, Tampa, USA

**Keywords:** bacteremia, edwardsiella tarda, infected pancreatic necrosis, intra-abdominal infection, liver cirrhosis, necrotizing pancreatitis, sepsis, source control

## Abstract

*Edwardsiella tarda* (*E. tarda*) is a rare human pathogen that most commonly causes self-limited gastroenteritis. Invasive infection, including bacteremia, is uncommon but carries substantial mortality, particularly among patients with underlying liver disease. We present a 78-year-old man with multiple comorbidities and previously undiagnosed cirrhosis who was admitted with altered mental status and sepsis. Computed tomography (CT) of the abdomen and pelvis demonstrated acute necrotizing pancreatitis with a large gas-containing peripancreatic collection. Blood cultures and intraoperative abscess cultures grew* E. tarda*, along with *Escherichia coli (E. coli)*. The patient underwent laparoscopic pancreatic necrosectomy and cholecystectomy and received piperacillin-tazobactam based on susceptibility testing. Blood cultures cleared after surgical intervention, and he improved clinically prior to discharge to a skilled nursing facility. This case highlights infected necrotizing pancreatitis as a rare intra-abdominal source of *E. tarda* bacteremia and reinforces the need for prompt source control in addition to appropriate antimicrobial therapy.

## Introduction

Necrotizing pancreatitis occurs in approximately 10%-20% of patients with acute pancreatitis and carries significantly increased mortality once secondary infection develops. Mortality increases from less than 10% in cases of sterile necrosis to 20%-30% once infection is present and can exceed 30% in patients who develop persistent organ failure [1,2]. Infected pancreatic necrosis is most commonly caused by enteric Gram-negative organisms such as *Escherichia coli *(*E. coli*),* Klebsiella *species, and *Enterobacter *species[1,2]. The isolation of an environmental organism such as *Edwardsiella tarda* (*E. tarda*) in this setting is therefore uncommon and highlights the potential for atypical pathogens to cause severe pancreatic infection [3-5].

*Edwardsiella tarda *is a motile, facultatively anaerobic Gram-negative bacillus found in aquatic environments and typically associated with exposure to contaminated seafood or freshwater sources [3,4,6]. While it most often presents as self-limited gastroenteritis, invasive disease has been reported, including bacteremia, hepatobiliary infection, soft tissue infection, and intra‑abdominal abscess formation. These presentations occur most frequently in patients with underlying hepatobiliary disease, diabetes, malignancy, or other immunocompromising conditions [3,5-7]. Importantly, invasive* E. tarda *infections are associated with high mortality, highlighting the pathogenic potential of this organism when identified in infected pancreatic necrosis [6-8].

## Case presentation

A 78-year-old man with a history of coronary artery disease status post percutaneous coronary intervention, atrial fibrillation on apixaban, chronic obstructive pulmonary disease, hypertension, hyperlipidemia, and gout presented to the emergency department with altered mental status. Owing to confusion, history was obtained primarily from his wife. Approximately one month before admission, he developed intermittent abdominal pain and experienced a single episode of nausea and vomiting two weeks before presentation. Over the subsequent 1-2 weeks, he had decreased oral intake, followed by progressive confusion beginning five days prior to admission. There were no reported fevers, chest pain, dyspnea, diarrhea, constipation, or persistent abdominal pain immediately preceding presentation.

He was a former smoker who quit in 1994 and consumed approximately two beers daily for >20 years. There was no known prior diagnosis of liver cirrhosis. He denied illicit drug use. His diet included frequent consumption of cooked fish without recent dietary changes. There was no history of raw or undercooked seafood ingestion, freshwater or saltwater exposure, recent travel, medication changes, or known sick contacts.

On arrival to the emergency department, the patient was hypotensive and tachycardic, with a heart rate of 100-133 beats per minute, respiratory rate of 28 breaths per minute, blood pressure of 87/52 mmHg (mean arterial pressure: 63 mmHg), oxygen saturation of 98% on room air, and temperature of 98.2°F. Electrocardiogram demonstrated atrial fibrillation with rapid ventricular response at approximately 130 beats per minute. Initial laboratory evaluation revealed leukocytosis with a white blood cell count of 29.8 × 10³/µL and a platelet count of 384 × 10³/µL. Serum lactate was 1.3 mmol/L, procalcitonin 0.22 ng/mL, creatinine 0.90 mg/dL, albumin 3 g/dL, and lipase 81 U/L. Serum ethanol level was <10 mg/dL. Blood pressure improved following intravenous fluid resuscitation, and vasopressor support was not required (Table 1).

**Table 1:** Initial laboratory findings on admission Laboratory values obtained at the time of hospital presentation with corresponding reference ranges

Laboratory test	Patient’s value	Reference range
White blood cell	29.8 th/uL	4.5-11.0 th/uL
Platelet count	384 th/uL	140-450 th/uL
Lactate	1.3 mmol/L	0.4-2.0 mmol/L
Procalcitonin	0.22 ng/mL	0.00-0.24 ng/mL
Creatinine	0.90 mg/dL	0.70-1.30 mg/dL
Albumin	3.0 g/dL	3.2-5.0 g/dL
Lipase	81 U/L	0-60 U/L
Serum ethanol	<10 mg/dL	0-10 mg/dL

**Figure 1:**
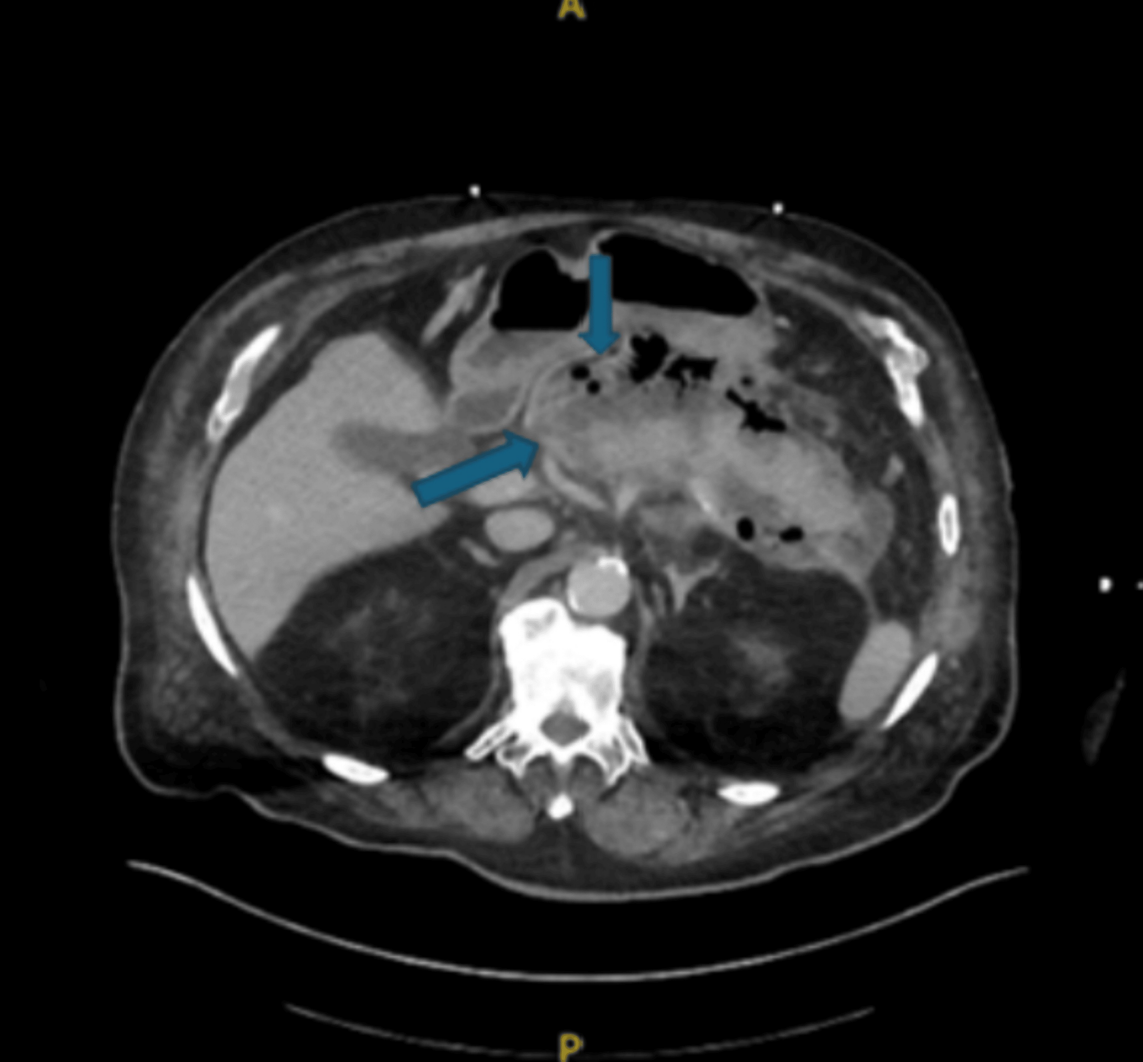
CT angiography of the abdomen and pelvis with and without contrast (axial view) showing multifocal areas of pancreatic necrosis: peripancreatic gas-containing fluid collection measuring up to 18.5 cm with the fluid collection abutting the greater curvature of the stomach and proximal small bowel loops CT: computed tomography

**Figure 2:**
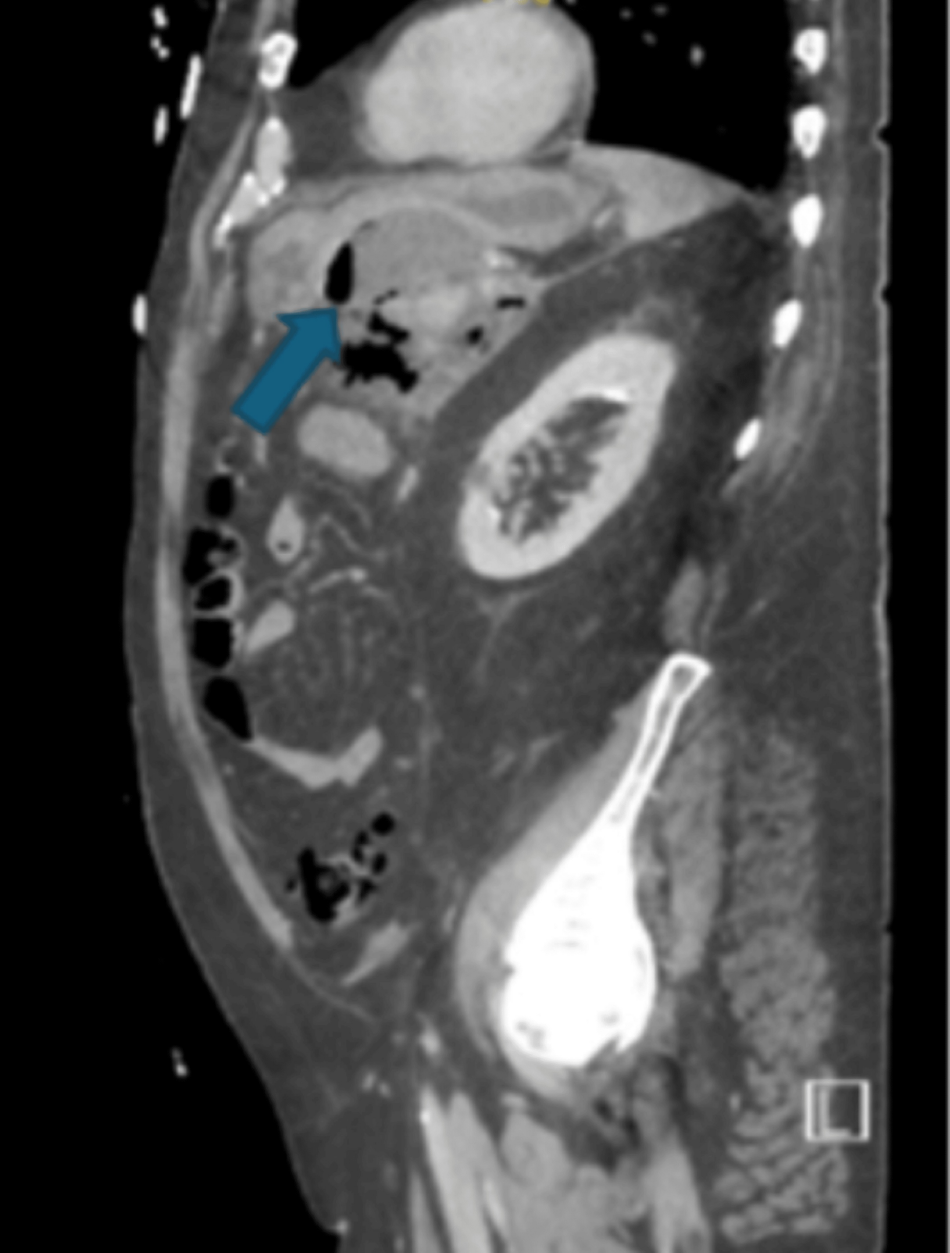
CT angiography of the abdomen and pelvis with and without contrast (sagittal view) showing pancreatic necrosis: peripancreatic gas-containing fluid collection measuring up to 18.5 cm with the fluid collection abutting the greater curvature of the stomach and proximal small bowel loops CT: computed tomography

Initial imaging, including chest radiograph, non-contrast computed tomography (CT) of the head, CT angiography of the chest, and bilateral lower extremity venous ultrasound, demonstrated no acute abnormalities. CT angiography of the abdomen and pelvis revealed acute necrotizing pancreatitis with a large gas-containing peripancreatic fluid collection measuring up to 18.5 cm, abutting the greater curvature of the stomach and proximal small bowel (Figure 1 and Figure 2). The liver demonstrated a lobulated contour consistent with cirrhosis, with associated small-volume ascites. Additional findings included cholelithiasis, colonic diverticulosis, and prostatomegaly.

The patient received intravenous fluid resuscitation with lactated Ringer’s solution and was empirically started on cefepime and vancomycin. He was transferred to a tertiary care center for hepatobiliary surgical evaluation.

Upon arrival in the intensive care unit, atrial fibrillation with rapid ventricular response recurred, with heart rates in the 130s. Intravenous metoprolol was initiated, and anticoagulation was held in anticipation of surgical intervention. Transthoracic echocardiography demonstrated normal left ventricular systolic function with an ejection fraction of 55%-60%, mild concentric left ventricular hypertrophy, and no pericardial effusion. Antimicrobial therapy was narrowed to piperacillin-tazobactam 4.5 g every 8 hours. 

On hospital day 2, the patient underwent laparoscopic pancreatic necrosectomy and cholecystectomy for infected necrotizing pancreatitis. Intraoperative exploration revealed a large, multiloculated peripancreatic cavity containing approximately 1 liter of purulent and necrotic material. Extensive debridement was performed until viable granulation tissue was encountered, and intraoperative cultures were obtained. Given the multiloculated collection without a safe endoscopic window, cystogastrostomy was not feasible. A 19-Fr Blake drain was placed in the debrided pancreatic bed. The patient tolerated the procedure well and returned to the intensive care unit, extubated on 2 liters of oxygen via nasal cannula.

**Figure 3:**
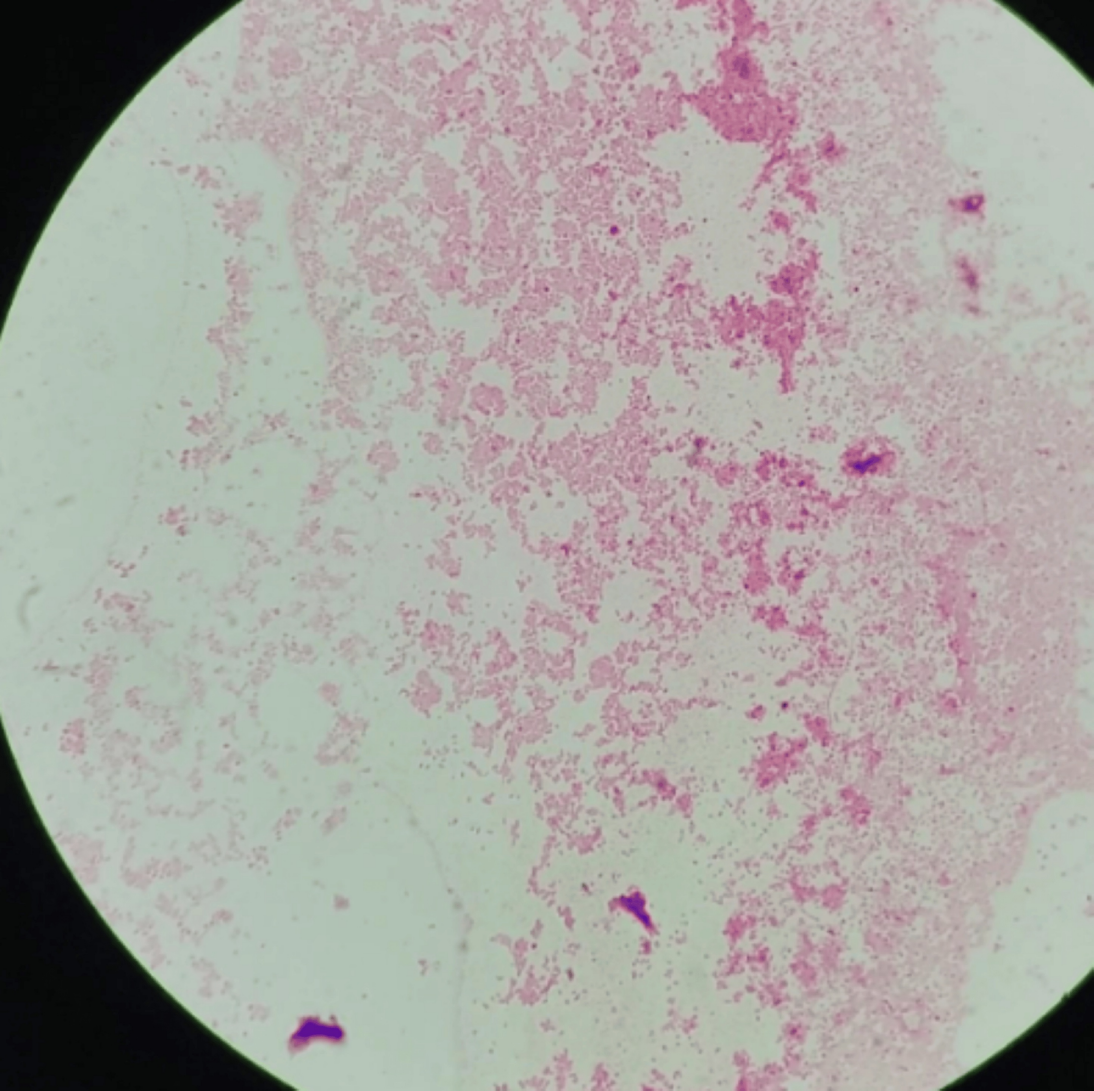
Gram stain of a positive blood culture Individual Gram-negative bacilli are not well visualized at this resolution; organism identification as *Edwardsiella tarda* was confirmed by culture growth on MacConkey agar as a non-lactose-fermenting Gram-negative bacillus and routine microbiologic confirmation.

Blood cultures obtained at admission grew Gram-negative rods in one aerobic bottle after 23 hours and were finalized as *E. tarda* within 72 hours (Figure 3). Antimicrobial susceptibility testing demonstrated pan-susceptibility, including sensitivity to piperacillin-tazobactam. Cultures from the peripancreatic abscess demonstrated heavy growth of *E. tarda* and *Escherichia coli* (*E. coli*), both susceptible to the ongoing regimen. Repeat blood cultures obtained 72 hours after admission were negative.

The patient demonstrated continued clinical improvement and was transferred out of the intensive care unit on hospital day 5. Heart rate remained controlled, and metoprolol was transitioned to bisoprolol. Apixaban was resumed on hospital day 7. He was discharged to a skilled nursing facility with a planned three-week course of intravenous piperacillin-tazobactam administered via a peripherally inserted central catheter, with scheduled follow-up in hepatobiliary surgery and infectious disease clinics.

## Discussion

*Edwardsiella tarda *is a rare human pathogen and an infrequent cause of extraintestinal infections [3,5]. The majority of documented human cases present as self-limited gastroenteritis [3,4]. Bacteremia and sepsis are rarely reported and, when present, especially in patients with pre-existing comorbidities, are associated with significant morbidity and mortality [6-9]. This is supported by the retrospective study and multi-language literature review by Hirai et al. in 2015, which reported an overall mortality of 44.6%, increasing to 61.1% among patients with soft tissue infections [6]. Among patients with documented *Edwardsiella tarda* bacteremia (ETB), comorbidities such as solid tumors, hepatobiliary disease, diabetes mellitus, chronic liver disease, and advanced age were commonly present [2,5,6]. Liver cirrhosis was independently associated with death (OR: 12.0) [6].

The hepatobiliary system is frequently reported as the primary source of ETB, with conditions such as cholecystitis, liver abscess, and cholangitis commonly implicated, in contrast to pancreatitis, which has only been mentioned in the literature as early as 1990 [3,8-10]. Yang and Wang described ETB associated with acute pancreatitis and pyomyoma, which was managed with antibiotic therapy and total hysterectomy with bilateral salpingo-oophorectomy [11]. The association between ETB and necrotizing pancreatitis is not well defined and is not an established primary infectious source [10].

*Edwardsiella tarda* may act as a saprophyte within the gastrointestinal tract; however, several findings in this case support its role as a true pathogen rather than incidental colonization in a polymicrobial environment. *Edwardsiella​​​​​​* *tarda *was isolated from normally sterile sites, including both blood cultures and peripancreatic abscess fluid. Microbiologic culture demonstrated growth on MacConkey agar as a non-lactose‑fermenting Gram‑negative bacillus, confirming viable organism recovery rather than incidental detection. Furthermore, the presence of a substantial gas‑containing peripancreatic collection on imaging, together with concordant isolation of *E. tarda* from blood and abscess cultures, strongly supports true pancreatic involvement rather than secondary bacteremia. Taken together, these findings broaden the recognized spectrum of deep intra‑abdominal sources of ETB and suggest that pancreatic necrosis may serve as a focal point for invasive infection in susceptible individuals.

Transmission of *E. tarda* classically follows ingestion of contaminated aquatic food or exposure to brackish water [3,4,6]. In the present case, the patient denied ingestion of raw or undercooked seafood, freshwater or saltwater exposure, recent travel, or occupational contact with aquatic environments, and reported only routine consumption of cooked fish without recent dietary changes. This absence of identifiable exposure is consistent with prior reports noting that many patients with *E. tarda* bacteremia lack a clear environmental or dietary source, raising the possibility of bacterial translocation in the setting of mucosal disruption or severe comorbidity [6,8,9]. Furthermore, unlike uncomplicated gastroenteritis, invasive* E. tarda* infection frequently presents without diarrhea and instead manifests with sepsis or focal infection [12].

Li et al. identified TraT, a surface-localized virulence determinant that facilitates complement resistance and cellular invasion [12]. In patients with cirrhosis, like ours, where innate immunity is impaired, TraT-mediated complement evasion provides a biologic explanation for the persistence of bacteremia and the high mortality observed in invasive *E. tarda* infection. In this context, infected pancreatic necrosis constitutes a plausible nidus for systemic spread.

Despite differing anatomic sites, invasive *E. tarda *infections share a common feature: involvement of necrotic or ischemic tissue. Mycotic aneurysms, necrotizing fasciitis, and necrotizing pancreatitis create environments characterized by tissue hypoperfusion, impaired host defenses, and increased local iron availability, all of which favor bacterial persistence and bloodstream invasion [13-16].

The patient’s atypical presentation, characterized by altered mental status, minimal gastrointestinal symptoms, and absence of fever, contributed to diagnostic complexity. In addition, the patient had no history of exposure to contaminated food or brackish water, suggesting an alternative route of infection. Elderly patients with underlying liver cirrhosis often present with nonspecific symptoms, making identification of the infectious source challenging; therefore, maintaining a high index of suspicion for deep-seated infections when ETB is detected is crucial to avoid rapid clinical deterioration and high mortality [6,8].

Management of ETB requires early initiation of broad-spectrum antimicrobial therapy in addition to definitive source control strategies. Isolates of *E. tarda* generally demonstrate susceptibility to β-lactams, cephalosporins, carbapenems, fluoroquinolones, and aminoglycosides, although intrinsic resistance to colistin and polymyxins has been described [3,4,6-8]. In our case, blood cultures and polymicrobial peripancreatic abscess cultures (*E. tarda* and *E. coli*) demonstrated pan-susceptibility to tested antibiotics, including piperacillin-tazobactam with a minimum inhibitory concentration of <4. Clinical improvement was achieved only after pancreatic necrosectomy and drainage, indicating that antimicrobial therapy alone may be insufficient without effective source control, particularly in patients with extensive necrotic disease [13-15].

To our knowledge, this represents the first reported case of *E. tarda *bacteremia arising from infected necrotizing pancreatitis, establishing pancreatic necrosis as a previously unrecognized deep intra-abdominal source of ETB. Reports of *E. tarda* infection involving mycotic aneurysms, necrotizing soft tissue infections, and, in our case, infected necrotizing pancreatitis suggest that this organism preferentially causes invasive disease when seeded into necrotic, ischemic tissue in cirrhotic hosts, where impaired immune response and limited antimicrobial penetration allow rapid progression to bacteremia and sepsis [6-8,13-16].

## Conclusions

This case highlights infected necrotizing pancreatitis as an uncommon but clinically significant intra‑abdominal source of *E. tarda* bacteremia. Lack of fever and the presence of previously unrecognized cirrhosis contributed to an atypical and diagnostically challenging presentation, underscoring the variable manifestations of invasive edwardsiellosis in older adults. When *E. tarda* bacteremia is identified, clinicians should maintain a high index of suspicion for deep‑seated intra‑abdominal infection, even in the absence of classic exposure history or gastrointestinal symptoms, particularly in patients with underlying liver disease. Early recognition, thorough source evaluation, and timely surgical source control, in conjunction with appropriate antimicrobial therapy, are critical to improving clinical outcomes in this high‑mortality infection.
